# Supplementary material for: Using the Theory of Planned Behavior to determine COVID-19 vaccination intentions and behavior among international and domestic college students in the United States
Source: PLoS One. 2024 Feb 2;19(2):e0293130. doi: 10.1371/journal.pone.0293130 (PMC10836687; doi:10.1371/journal.pone.0293130)
Supplement: Attachment — Submitted filename: Review- PONE-D-23-12951.pdf [file pone.0293130.s003.pdf]

Review- PONE-D-23-12951 **- Using Theory of Planned Behavior to Determine COVID-19 Vaccination Intention and Behavior among International and Domestic College Students in the United States**

Reviewer**: Phan Minh Trang, MD. PhD**

The meaning of study

The global pandemic covid 19 has caused many serious consequences with millions of deaths around the world between 2020 and 2023. Covid19-vaccination has got great effectiveness in preventing the virus transmission as well as high mortality in humans.

Using the TPB (theory of planned behavior (TPB) including attitude, behavioral intention, perceived behavioral control, and subjective norms, a robust model is highly predictive of human actions in specific contexts. In this paper, research team investigated whether attitude, behavioral intention, PBC, and subjective norms were associated with full COVID-19 vaccination and the intention to receive a booster among international and domestic college students in the United States. The result of study indicated the behavior intention to vaccinate covid 19 among students both international and domestic in the United States in which students' intentions to receive COVID-19 vaccines significantly predicted their intentions to receive a booster, with international students scoring higher on booster intentions. Among the combined college student population, attitude, intention to receive COVID-19 vaccines, and subjective norms each significantly predicted students' intentions to receive a booster. This finding has helped health managers more information to develop health policies to prevent covid 19 infection.

However, there are several limitations of the paper such as the sample size, instrument development including internal consistency, construct validity, predictive validity assessment, ... of research questionnaire. The authors need to clarify study methodology so that readers can understand more easily about the reliability and accuracy of these research results.

Abstract

In this abstract, there are 241-word to briefly present the overview, study aim as well as subjects, data analysis methods, typical results of the study, and conclusions.

Introduction

In this part, the authors summarized the prevalence of covid-19 infection in the US and the benefits of covid-19 vaccination. In addition, there have had some studies on the awareness and attitude of covid-19 vaccination among young people including students at colleges in which the rate of covid-19 vaccination in this group is still low due to many reasons such as distrusting vaccines, worrying its side-effects, believing in natural immunological system, etc. Especially, applying the theory of planned behavior (TPB), including attitude, behavioral intention, perceived behavioral control (PBC), and subjective norms to search for an answer as to whether psychosocial factors are involved with covid 19 vaccination among domestic and international college students.

Theoretical Framework

Although the author mentioned the TPB theory by using the tool to research on the attitudes and behaviors of students in vaccination against influenza and HPV, the study aim in this paper was the attitudes and behaviors of students in vaccination against the covid-19 virus, a very new, causing a pandemic, unknown side effects of the vaccine, ... TPB of covid-19 vaccination is very different from that of vaccination against influenza or HPV, an age-old vaccine and its effectiveness as well as side effects have been proven. Therefore, *the author should present the theoretical part of TPB on vaccination against covid 19 in which attitude, behavior intention, subjective norms, and PBC are related to covid-19 vaccination*.

Method and materials

In this part, it is good at that author presented variables such as attitude with possible scores (7 – 49) and Cronbach' alpha value (0.93 for international students and 0.89 for domestic students); behavioral intention with possible scores (3 – 21) and Cronbach' alpha value (0.98 for international students and 0.92 for domestic students); PBC with possible scores (6 – 42) and Cronbach' alpha value (0.85 for international students and 0.86 for domestic students); subjective norms with possible scores (4 – 28) and Cronbach' alpha value (0.94 for international students and 0.82 for domestic students). Moreover, binary logistic regression and hierachical multiple linear regression were applied to analyse data.

However, authors should clarify and present more information:

- *The way to choose sample size in which how many international and domestic students at colleges in the USA are studying. For example: there are 1,1 million international students in USA, but a sample size of 197 students were counted in the study.*

- *The way to develop the TPB-based instrument includes pilot studies of qualitative and quatitative, expert panel review, pilot test, test retest, internal consistency, construct validity, and predictive validity assessment.*
- *An exploratory factor analysis (EFA) is used to determine the number of factors and percentages of explained variance which extracted factors according to eigenvalues greater than 1.0, factor loading higher than 0.5, and used processes of principal components factor and orthogonal rotation. In addition, confirmatory factor analysis (CFA) is applied to evaluate the validity of the health belief items. A value of factor item loadings at or above the 0.3 level was considered significant in factor analysis.*

- *The goodness-of-fit indices were used to assess the observed model and the theoretical model or the final structural model exhibited acceptable fit of the data*

- *Explaining more clearly the block 1 and block 2, are they the same model 1 and model 2?*

Results

- Research results are interpreted in 4 main points (participant characteristics, TBP-based psychosocial factors, determinants of receiving full COVID-19 vaccines, and determinants of intention to receive a COVID-19 booster) with tables 1- 4. This result answers the objective and hypothesis study.

- *However, before presenting results, author should interpret the stages of the TPB-based instrument development including pilot studies of qualitative and quantitative, expert panel review, pilot test, test retest, internal consistency, construct validity, and predictive validity assessment.*

Discussion

In the discussion, the author should discuss on the study results from 4 main points in the results section. It should be presented more concisely, focusing on specific results answering the study aim of the paper.

References

Author should choose a same way to make references, *for example*: reference (4) at page 3 different from other references.

Appendix

- *Lack of questionnaires as a TPB-based instrument*
- *Lack of tables and figures is related to the TPB-based instrument development involving pilot studies of qualitative and quantitative, expert panel review, pilot test, test retest, internal consistency, construct validity, and predictive validity assessment.*
