# Supplementary material for: Using the Theory of Planned Behavior to determine COVID-19 vaccination intentions and behavior among international and domestic college students in the United States
Source: PLoS One. 2024 Feb 2;19(2):e0293130. doi: 10.1371/journal.pone.0293130 (PMC10836687; doi:10.1371/journal.pone.0293130)
Supplement: Attachment — Submitted filename: PLOS ONE_reviewer.doc [file pone.0293130.s002.doc]

To be accepted for publication in PLOS ONE, research articles must satisfy the following criteria:

**1. The study presents the results of original research.**

Yes. The manuscript presents an insightful investigation into the factors influencing COVID-19 vaccination uptake among two distinct groups of international and domestic students. The study compares and contrasts the elements of the Theory of Planned Behavior (TPB) as predictors of vaccination behavior and booster receival between these 2 student groups in the context of the United States.

Implications for Public Health Interventions: The manuscript appropriately discusses the practical implications of the findings. The identification of specific factors associated with Covid-19 vaccination, such as perceived behavioral control and subjective norms, provides valuable guidance for designing targeted interventions and strategies to promote vaccination uptake among both international and domestic students.

**2. Results reported have not been published elsewhere.**

The findings presented in this study have not been previously published or reported in any other source.

**3. Experiments, statistics, and other analyses are performed to a high technical standard and are described in sufficient detail.**

Inclusion of Relevant Variables: The manuscript takes into account of TPB elements (attitude, behavioral intention, perceived behavioral control, and subjective norms) and important factors beyond TPB variables, such as chronic illnesses and smoking habits, to better understand their associations with COVID-19 vaccination. This comprehensive approach strengthens the analysis and provides a more holistic view of the determinants of vaccination uptake among college students.

Methodological Rigor: The study employs a robust methodology, utilizing a binary logistic regression analysis to examine the relationship between TPB variables (attitude, behavioral intention, perceived behavioral control, and subjective norms) and COVID-19 vaccination and booster acceptance among international and domestic students. This approach enhances the reliability and validity of the findings.

Comparative Analysis: By comparing the responses and vaccination rates of international and domestic students, the study reveals significantly higher vaccination rates among international students and provide valuable insights into the influence of contextual factors such as government policies on vaccination behaviors. Moreover, this comparison reveals disparities in determinants of COVID-19 vaccine and booster uptake between the two groups, aiding the design of targeted interventions for international and domestic students.

**4. Conclusions are presented in an appropriate fashion and are supported by the data.**

Conclusions are presented in a clear outline of 2 sections pertaining to the summary of the findings and implications for health interventions. However, there are still places for improvement. The conclusion only presented a part of the results through the wrap-up of associated factors with vaccination only among domestic college students and all students. Those findings relating to international students should be summarized and key differences between 2 groups should be highlighted. Based on this, the author might emphasize the need for health interventions that are exclusive for each group. Such practical implications will provide more actionable insights for policymakers, healthcare professionals, and educators.

**5. The article is presented in an intelligible fashion and is written in standard English.**

Although the study utilizes a robust methodology that yields important and relevant findings, the presentation of the discussion lacks clarity and coherence. The discussion elaborates the relationship between psychosocial elements of the TPB (i.e., attitude, behavioral intention, perceived behavioral control, and subjective norms) and full COVID-19 vaccination, as well as intentions to receive a booster, in both domestic and international students. However, the information pertaining to the associated factors with full vaccination or the booster is intertwined throughout the discussion, making it difficult to distinguish between them. Similarly, the information describing the determinants among domestic and international students is mixed within paragraphs, hindering the viewers' ability to discern the key differences. Seemingly, determinants of full Covid-19 vaccination among domestic students include PBC, chronic illnesses and smoking. Regarding the reception of boosters in domestic students, the intention to receive the COVID-19 vaccine predicts the intention to receive the booster. However, viewers might have difficulty identifying associated factors as these pieces of information were scattered throughout the discussion. Furthermore, there is repetition of certain points in discussion, further impeding its clarity and coherence. Several repeated points and their location are listed below:

**Paragraph 1:** *International students COVID-19 vaccination rates were significantly higher than for domestic students.* **Later in paragraph 1:** *More international students (81.2%) than domestic students (55%) in our sample had already received COVID-19 vaccines*

**Paragraph 5:** *PBC significantly predicted domestic students’ full vaccination, reflecting confidence in their ability to be vaccinated against COVID-19.* **Paragraph 9:** *PBC was the sole TPB variable to significantly predict vaccination against COVID-19 among domestic students and the combined student sample in this study.*

To enhance the clarity and cohesiveness of the discussion, I suggest a thorough rewrite in which a clear outline of TBP associated factors and primary determinants of full covid-19 vaccination as well as booster acceptance should be provided in 2 separate sections dedicating to international and domestic students. To improve the overall readability of the discussion, the author should avoid repetition and unnecessary redundancy.

**6. The research meets all applicable standards for the ethics of experimentation and research integrity.**

Yes

**7. The article adheres to appropriate reporting guidelines and community standards for data availability.**

Yes
