# Supplementary material for: Using the Theory of Planned Behavior to determine COVID-19 vaccination intentions and behavior among international and domestic college students in the United States
Source: PLoS One. 2024 Feb 2;19(2):e0293130. doi: 10.1371/journal.pone.0293130 (PMC10836687; doi:10.1371/journal.pone.0293130)
Supplement: Attachment — Submitted filename: Responses to reviewers comments HL 8.7.2023.docx [file pone.0293130.s004.docx]

Dear Editor,

Thank you very much for providing us with the great opportunity to revise and resubmit our manuscript. We have addressed every reviewer’s comment in the revised manuscript and highlighted all changes in yellow. We also revised our manuscript based on Journal Requirements.

Table 1. Responses to reviewer 1 Dr. Phan Minh Trang, MD., Ph. D. comments

| Comments | Responses |
| --- | --- |
| 1. For theoretical framework, Dr. Trang mentioned that the author should present the theoretical part of TPB on vaccination against COVID-19 in which attitude, behavior intention, subjective norm, and PBC are related to COVID-19 vaccination. | Dear Dr. Trang, thank you for your suggestion. We revised the theoretical framework and added more information to support how TPB constructs are related to COVID-19 vaccination on Page 6 Line 126-130. |
| 2. Regarding methods and materials, authors should clarify and present more information:  - The way to choose sample size in which how many international and domestic students at colleges in the USA are studying. - The way to develop the TPB-based instrument includes pilot studies of qualitative and quantitative, expert panel review, pilot test, test retest, internal consistency, construct validity, and predictive validity assessment. - An exploratory factor analysis (EFA) is used to determine the number of factors and percentages of explained variance which extracted factors according to eigenvalues greater than 1.0 factor loading higher than .05 and used process of principal component factor and orthogonal rotation. The goodness-of-fit indices were used to assess the observed model and the theoretical model or the final structural model exhibited acceptable fir of the data. - Explaining more clearly block 1 and block 2, are they the same model 1 and model 2. | Details were added to describe the procedures for determining sample size for each group. (Page 7 Line 152-159). We added “Using the G*Power program, we assumed a small effect size with an odds ratio (OR) of 1.68, 50% probability of not being fully vaccinated and a significance level of 0.05, a sample size of 133 was needed to achieve at least an 80% study power when using logistic regression models to examine factors related to the full COVID-19 vaccination status. With a predicted 15% missing data rate, we over sampled to obtain at least 157 participants in each group. This sample size was also adequate for achieving a minimum of 80% study power when using hierarchical linear regression models to examine 17 variables that might be associated with intentions to receive a COVID-19 booster.”  On Page 8 Line 161-171, we described that we adapted the instrument from Dr. Catalano’s instrument by replacing ‘HPV vaccine’ with ‘COVID-19 vaccine.’ We added more details about the procedures and psychometric properties of the original instrument used to assess TPB constructs among college students in the revised manuscript. We added “Study measures were adapted from the TPB-based instrument developed by Catalano and colleagues. The original version was developed to assess attitudes, PBC, subjective norms, and behavioral intentions in the context of HPV vaccination among college students. The original instrument demonstrated acceptable readability for college students, good face and content validity via an expert panel review, acceptable test-retest reliability, and great internal consistency reliability with Cronbach’s alphas ranging from 0.92 to 0.97. In addition, the original instrument had good construct validity through factor loadings of four constructs and acceptable predictive validity explaining 58% of the variance in intentions to receive HPV vaccine [16]. We adapted this instrument by replacing ‘HPV vaccine’ with ‘COVID-19 vaccine,’ while maintaining the same question items, to measure attitudes, PBC, subjective norms, and behavioral intentions in relation to receiving COVID-19 vaccines and boosters.”  We also evaluated the internal consistency reliability of the adapted instrument among international and domestic college students in our study and results showed great reliability (Page 13 Line 255-265).  We added “**Construct validity**. Results from the factor analysis support the good construct validity of the adapted instrument. The KMO was 0.91 indicating sampling adequacy [20]. Bartlett’s test of sphericity was statistically significant (*p* < .001). Four factors were extracted and explained 69.35% of the total variance in Table 2.  **Internal consistency reliability**. Cronbach’s alpha was employed to assess internal consistency in this study. The Cronbach’s alpha for attitudes toward behavior subscale was .93 among international students and .89 among domestic students. For the PBC subscale, it was .85 for international students and .86 for domestic students. The Cronbach’s alpha for subjective norms subscale was .94 for international students and .82 for domestic students. For the behavioral intentions subscale, Cronbach’s alpha was .98 for international students and .92 for domestic students.”  To further examine the construct validity of the revised instrument in our study, we conducted exploratory factor analysis. Factor loadings and average explained variance were provided in Table 2. Results demonstrate great construct validity of the revised instrument used in the study. More details were added on Page 10 Line 214-218 and Table 2. We added “Construct validity of the TBP-based instrument was assessed employing Principal Components with varimax rotation analysis. The numbers of factors were determined by Kaiser–Meyer–Olkin (KMO), scree plots, loadings of over .40, and explainable percentages of variance [19]. Cronbach’s alpha was used to estimate the internal consistency reliability.”  Thank you for pointing this out. Yes, model 1 and model 2 are the same as block 1 and block 2. We changed from model 1 and model 2 to block 1 and block 2 to reduce confusion (Page 10 & 16 Line 228 & 229, 297 & 301). |
| 3. Regarding results, Dr. Trang mentioned that before presenting results, authors should interpret the stage of the TPB-based instrument development. | We adapted the instrument from Dr. Catalano and her research team. We addressed this on Page 7 Line 161-171. We also revised the paragraph to enhance clarity. |
| 4. For Discussion, Dr. Trang mentioned that we should present our findings more concisely on specific results answering the study aim of the paper | Following the suggestion, we revised the entire Discussion and also provided sub-headings to enhance clarity. |
| 5. Regarding References, Dr. Trang addressed that authors should choose the same way to make references. For example, reference (4) at page 3 is different from other references. | We apologize for the inconsistency. We carefully reviewed all the in-text citations and the reference list to make sure they are consistent and in the same formatting. |
| 6. Regarding Appendix, lack of the instrument and the table and figures related to the TPB-based instrument. | We adapted the instrument from Dr. Catalano and her research team. We addressed this on Page 7 Line 161-171. We also attached the instrument we utilized as Table 2. We added “Special thanks to Dr. Catalano for letting us utilize the instrument in our study” on Page 25 Line 483-484. |

Table 2. Responses to reviewer 2

| 1. For Conclusion—Conclusions are presented in an appropriate fashion and are supported by the data. Those findings relating to international students should be summarized and key differences between two groups should be highlighted. Based on this, the author might emphasize the need for health interventions that are exclusive for each group. | Thank you for your suggestion. We added more information related to health interventions on Page 24 Line 462-477. We added “PBC was a significant factor related to the full vaccination status among domestic college students, while attitudes were a significant factor associated with all students’ full vaccination status. Moreover, attitudes towards receiving a COVID-19 booster, subjective norms, and intentions to receive COVID-19 vaccines were significantly correlated with the intentions to receive a booster. From a theoretical perspective, our findings somewhat support that the TPB is an applicable framework for explaining COVID-19 vaccination intentions and behavior among both domestic and international college students. Thus, public awareness and educational programs aimed at promoting vaccine acceptance should consider using TPB as a framework and tailor to each group. To enhance uptake of COVID-19 vaccines and boosters and to reduce the incidence of severe cases, healthcare providers and educators can develop vaccine campaigns to promote COVID-19 vaccination among college students. For example, universities and colleges can provide bilingual health interpreters, implement campus-based marketing strategies, send reminders using social media, and offer free and affordable vaccines to increase vaccination rates among international students. When promoting the vaccination rate of domestic students, healthcare providers and educators need to understand students’ health concerns and answer their questions with evidence to increase their PBC.” |
| --- | --- |
| 2. For Discussion—The information describing the determinants among domestic and international students is mixed within paragraphs…To enhance the clarity and cohesiveness of the Discussion, I suggest a thorough rewrite in which a clear outline of TPB associated factors and primary determinants of full COVID-19 vaccination as well as booster acceptance should be provided in two separate sections. | We rewrote the entire Discussion by separating content on full COVID-19 vaccination from booster shot and providing sub-headings to enhance the clarity. |

Table 3. Responses to editor

| 1. PBC abbreviate for what is abstract | PBC is perceived behavioral control, one construct of the theory of planned behavior. We deleted the abbreviation and used the full term in abstract. |
| --- | --- |
| 2. Please explain that why author use median of the four TPB factors for making the cutoff that divide respondents into low-and-high scoring groups. | We chose the median as cutoff based on prior literature indicating that median cutoff of TPB constructs had high specificity; however, we deleted the information and re-did the binary logistic regression using continuous variables. Please see Page 10 Line 22-223. We revised it to “All covariates were dummy coded except age. The four TPB factors were analyzed as continuous variables.” |
| 3. Please have clear definition of variables that combined some criteria such as chronic diseases, smoking…and also table 2 intended vaccination COVID-19, intended to take booster (Table 2: Received 1 dose; fully vaccinated; received booster) that make confuse. | The definitions of COVID-19 vaccination status, health status (chronic diseases), and smoking status were added on Page 8 Line 172-183. We added “Respondents’ demographic information (e.g., age, sex, ethnicity, education, health status, smoking history, other vaccination status) was also collected. For health status, participants were asked to answer if they were currently diagnosed with any diseases including diabetes, hypertension, autoimmune disease, hepatitis asthma, and cardiac disease; had a chronic medical condition requiring medication or regular physician visits; or had overall very good health with no disease. For smoking status, participants were asked to identify if they smoked, never smoked, or used to smoke but quit. Participants were also asked to identify if they had received one dose of the COVID-19 vaccine, they were fully vaccinated including one dose of Johnson & Johnson or two doses of Pfizer/Moderna, they had already received a booster shot, or they never received any COVID-19 vaccines. For general vaccination, participants were asked to indicate how up-to-date they were on vaccinations, with responses including: currently up-to-date, no but planning to be updated, or no and not planning to be updated.”  We also added Notes “For definitions of COVID-19 vaccines, please refer to Measures.” for Table 4 and “For definitions of chronic illness (health status), updated vaccination, and smoking status, please refer to Measures” for Table 5. |
| 4. Please report the result of OR with 95% CI. | We added the 95% CI for the OR in the revised results section and Table 4. |
| 5. Please explain clear about “Domestic students who were male (OR: 3.22, p=.001) and had lower PBC (OR: 8.33, p=.049) had lower odds of being fully vaccinated” | We revised the sentence to “Domestic students who were female (OR = .31, 95% CI: .15-.63; *p* ≤ .001) and had higher PBC (OR = .12, 95% CI: .02-.99, *p* = .049) had lower odds of being fully vaccinated, consistent with our findings when combining both student groups.” on Page 15 Line 28-292. |
| 6. Please explain that why author use determinants instead of risk for OR? | To more accurately describe the results while considering the limitation of cross-sectional data, we revised our language to focus on “correlates” and “TPB-based factors.” When interpreting OR, we compared the odds but not risks between groups, as OR is not relative risk. |
| 7. Please explain that TPB model is appropriate to measure intended to take booster vaccine COVID-19 when R=0.08? Whether TPB model appropriate to all participants such as LMIC? They familiar with HBM than TPB. | R^2^ were 0.08, 0.15, and 0.08 for international, domestic, and all students in block 1 when including only demographic factors; however, R^2^ were 0.65, 0.72, and 0.68 in block 2 after adding the TPB-based factors (Table 5). The TPB explained a respectable portion (0.57, 0.57, 0.60) of variance in the intentions to receive a COVID-19 booster (Page 18 Line 318-320). We further discussed the application of the TPB in the context of vaccination for both domestic and international students in the revised discussion section. |
