# Supplementary material for: Using the Theory of Planned Behavior to determine COVID-19 vaccination intentions and behavior among international and domestic college students in the United States
Source: PLoS One. 2024 Feb 2;19(2):e0293130. doi: 10.1371/journal.pone.0293130 (PMC10836687; doi:10.1371/journal.pone.0293130)
Supplement: Attachment — Submitted filename: PONE reviewer2_phamduonguyenbinh.docx [file pone.0293130.s005.docx]

| Reviewer 2 comments | Author’s revision | Reviewer’s comments after revision |
| --- | --- | --- |
| For Conclusion—Conclusions are presented in an appropriate fashion and are supported by the data. Those findings relating to international students should be summarized and key differences between two groups should be highlighted. Based on this, the author might emphasize the need for health interventions that are exclusive for each group. | Thank you for your suggestion. We added more information related to health interventions on Page 24 Line 462-477. We added “PBC was a significant factor related to the full vaccination status among domestic college students, while attitudes were a significant factor associated with all students’ full vaccination status. Moreover, attitudes towards receiving a COVID-19 booster, subjective norms, and intentions to receive COVID-19 vaccines were significantly correlated with the intentions to receive a booster. From a theoretical perspective, our findings somewhat support that the TPB is an applicable framework for explaining COVID-19 vaccination intentions and behavior among both domestic and international college students. Thus, public awareness and educational programs aimed at promoting vaccine acceptance should consider using TPB as a framework and tailor to each group. To enhance uptake of COVID-19 vaccines and boosters and to reduce the incidence of severe cases, healthcare providers and educators can develop vaccine campaigns to promote COVID-19 vaccination among college students. For example, universities and colleges can provide bilingual health interpreters, implement campus-based marketing strategies, send reminders using social media, and offer free and affordable vaccines to increase vaccination rates among international students. When promoting the vaccination rate of domestic students, healthcare providers and educators need to understand students’ health concerns and answer their questions with evidence to increase their PBC.” | I have reviewed the revised manuscript. I agree that the author has added the findings relating to international students and emphasize the health intervention tailored to each group of students |
| For Discussion—The information describing the determinants among domestic and international students is mixed within paragraphs...To enhance the clarity and cohesiveness of the Discussion, I suggest a thorough rewrite in which a clear outline of TPB associated factors and primary determinants of full COVID- 19 vaccination as well as booster acceptance should be provided in two separate sections. | We rewrote the entire Discussion by separating content on full COVID-19 vaccination from booster shot and providing sub-headings to enhance the clarity. | I agree that the discussion was revised thoroughly and its clarity was enhanced greatly. I could see no repetition of findings in the discussion. The TBP factors associated with Covid-19 full vaccination and boosters receival were indeed separated into 2 sections with sub-headings. In each section, findings relating to international and domestic students were also presented. |
